# The effects of information and social conformity on opinion change

**DOI:** 10.1371/journal.pone.0196600

**Published:** 2018-05-02

**Authors:** Daniel J. Mallinson, Peter K. Hatemi

**Affiliations:** 1 School of Public Affairs, The Pennsylvania State University - Harrisburg, Middletown, Pennsylvania, United States of America; 2 Department of Biochemistry, The Pennsylvania State University, University Park, Pennsylvania, United States of America; 3 Department of Molecular Biology, The Pennsylvania State University, University Park, Pennsylvania, United States of America; 4 Department of Political Science, The Pennsylvania State University, University Park, Pennsylvania, United States of America; Southwest University, CHINA

## Abstract

Extant research shows that social pressures influence acts of political participation, such as turning out to vote. However, we know less about how conformity pressures affect one’s deeply held political values and opinions. Using a discussion-based experiment, we untangle the unique and combined effects of information and social pressure on a political opinion that is highly salient, politically charged, and part of one’s identity. We find that while information plays a role in changing a person’s opinion, the social delivery of that information has the greatest effect. Thirty three percent of individuals in our treatment condition change their opinion due to the social delivery of information, while ten percent respond only to social pressure and ten percent respond only to information. Participants that change their opinion due to social pressure in our experiment are more conservative politically, conscientious, and neurotic than those that did not.

## Introduction

Information and persuasion are perhaps the most important drivers of opinion and behavioral changes. Far less attention, however, has been given to the role of social pressure in opinion change on politically-charged topics. This lacuna is important because humans have a demonstrated proclivity to conform to their peers when faced with social pressure. Be it in the boardroom or on Facebook, Solomon Asch and Muzafer Sherif’s classic studies hold true today. Individuals conform based on a desire to be liked by others, which Asch [1, 2] called compliance (i.e., going along with the majority even if you do not accept their beliefs because you want to be accepted), or a desire to be right, which Sherif et al. [3] termed private acceptance (i.e., believing that the opinions of others may be more correct or informed than their own). These two broad schemas encompass many specific mechanisms, including, motivated reasoning, cognitive dissonance, utility maximization, conflict avoidance, and pursuit of positive relationships, among others. Information-based social influence and normative social influence (i.e., conformity pressure) both play important, albeit distinct, roles in the theories of compliance and private acceptance (see [4]). In both cases, humans exhibit conformity behavior; however only in private acceptance do they actually update their beliefs due to the social delivery of new information.

Extensions of Asch and Sherif’s path-breaking works have been widely applied across a number of behavioral domains [5–9], to include political participation. For example, significant attention has been focused on the import of conformity on voter turnout and participatory behaviors [10], including the effects of social pressure on the electoral behavior of ordinary citizens [11–15]. This body of work points to both the subtle and overt power of social influence on electoral behavior, yet little is known about the import of social conformity for politically charged topics in context-laden circumstances, particularly those that challenge one’s values and opinions.

Testing conformity pressure in the ideological and political identity domain may explicate whether the pressure to align with an otherwise unified group is different when dealing with politically charged topics versus context-free topics such as the size of a line or the movement of a ball of light [2, 16]. Opinions on politically charged topics are complex, value laden, aligned with cultural norms, and not easily changed [17–21]. It remains unknown if the effects of social conformity pressures on political opinions are conditioned by the personal nature of the locus of pressure. To be sure, social conformity is a difficult concept to measure without live interaction. An observational approach makes it difficult to untangle if or how social pressure independently affects behaviors given these variegated casual mechanisms, and whether changes in opinion that result from social interaction are due to compliance or private acceptance. Nevertheless, experiments provide one means to gain insight into how and why opinion change occurs. Here, we undertake an experiment to test the extent to which opinion change is due to persuasion through new information, social conformity pressure, or a combination of the two in a more realistic extended discussion environment.

## Conformity and political behavior

Both observational and experimental research has addressed different aspects of the impact of socially-delivered information on individual behavior. Observational analyses of social networks form the backbone of much of the recent research on social influence and political behavior. Sinclair [22], for instance, demonstrates that citizen networks convey a bounded set of political information. Individuals may turn to highly informed peers [23] or aggregate information from trusted friends and family [24] in order to reduce the cost of gathering the information required to engage in political behavior (e.g., voting). In turning to their network, they are open to privately accepting this useful information. Political information, however, is not the only type of information transmitted through personal networks. Social pressure helps the network induce compliance with desired social norms [25–27]. In this case, members of the network provide information regarding the group’s expectations for appropriate engagement in politics. Individuals that are concerned about whether or not the group will continue to accept them therefore conform out of a desire to be liked, broadly defined. Norms are often self-enforcing, with merely the perceived threat of potential sanctions being enough to regulate behavior through compliance and self-sanctioning [28, 29].

The debate over the practicality and reality of deliberative democracy further highlights the importance of understanding the role of political conformity in public and elite discourse. Scholars and theorists argue that political decisions are improved and legitimized under a deliberative process [30–34], even though deliberation does not necessarily result in consensus [35]. The crux of democratic deliberation is that participants are engaging in a rational discussion of a political topic, which provides the opportunity for each to learn from the others and thus privately update their preferences (i.e., out of a desire to be right). It results in a collectively rational enterprise that allows groups to overcome the bounded rationality of individuals that would otherwise yield suboptimal decisions [36]. This requires participants to fully engage and freely share the information that they have with the group.

Hibbing and Theiss-Morse [37], however, raise important questions about the desirability of deliberation among the public. Using focus groups, they find that citizens more often than not wish to disengage from discussion when they face opposition to their opinions. Instead, they appear averse to participation in politics and instead desire a “stealth democracy,” whereby democratic procedures exist, but are not always visible. In this view, deliberative environments do not ensure the optimal outcome, and can even result in suboptimal outcomes. In fact, the authors point directly to the issue of intra-group conformity due to compliance as a culprit for this phenomenon. The coercive influence of social pressure during deliberation has been further identified in jury deliberations [38, 39] and other small group settings [40].

Beyond politics, there is experimental evidence of the propensity to conform out of a desire to either be liked or to be right [25, 41–45]. Using a simple focus group format and pictures of lines, Asch [1, 2] demonstrated that individuals would comply with the beliefs of their peers due to a desire to be accepted by the group, even if they disagree and even when they believe the group opinion does not match reality. To do this, Asch asked eight members of a group to evaluate two sets of lines. The lines were clearly either identical or different and group members were asked to identify whether there was a difference. Unknown to the participant, the seven other group members were confederates trained to act in concert. At a given point in the study, the confederates began choosing the wrong answer to the question of whether the lines were equal. Consequently, the participant faced social pressure from a unified group every time they selected their answer. Asch varied the behavior of the group, including the number of members and number of dissenting confederates. Participants often exhibited stress and many eventually complied with the group consensus, even though the group was objectively wrong and participants did not agree with them privately.

Using a much more complex and context-laden format—a youth summer camp with real campers—Sherif et al. [3] demonstrated private acceptance whereby humans internalize and conform to group norms because consensus suggests that they may have converged on a right answer. In this case, the boys in the camp quickly coalesced into competing factions and initial outliers in the groups conformed out of a desire to win competitions (i.e., be right). While the groundbreaking Robbers Cave experiments revealed a great deal about group behavior well beyond conformity, we focus specifically on this particular aspect of the findings, which have stood the test of time in numerous replications and extensions across a wide variety of social domains [46–52].

Replication of Asch’s experimental work, in particular, has met varying degrees success. Lalancette and Standing [53]found that Asch’s results were mixed when using a prompt more ambiguous than unequal lines. Further, Hock [54] critiques the Asch design for not replicating a real life situation. Focusing on divorce attitudes, Kenneth Hardy provided an early application of Asch’s public compliance and Sherif’s private acceptance theories to political opinions using a similar small-group format with six confederates and one participant. Confederates offered not only their opinions, but also reasons for their opinions, which provided a methodological innovation by introducing more information than just the confederates’ votes. Hardy’s work provided an important starting point for identifying the process of conformity in the political realm, but it remains limited. He only utilized men in his study and did not allow for repeated discussion to assess how long participants hold up to conformity pressure. In a more recent study, Levitan and Verhulst [55]found persistence in political attitude change after interaction with a unanimously-opposing group, but they did not incorporate any discussion.

Our experiment builds on these works by examining the micro-process underlying opinion change for a politically charged topic discussed in a real context. We bridge between studies that allow for no discussion with those that study day-long deliberations in order to determine if group influence has a stronger effect, even when the discussion centers on an attitude closely tied with social identity. Our interaction of about an hour simulates a likely real-world example of dialogue. More importantly, our design allows us to speak to the debate over social influence by pulling apart the desires to be right (private acceptance) and liked (compliance). Our main goal is not to completely predict the general public’s behavior, but rather to identify the independent causal role of social pressure on opinion change, given the known import of information effects. We expect conformity pressure and information to have joint and independent effects on opinion change.

### Variation in conformity behavior

While our primary interest is in identifying the average effects of information and conformity pressure on opinion change, we nevertheless recognize that there is variation in humans’ responses to social pressure, depending on observed and unobserved individual characteristics. Thus the average treatment effect recovered can mask substantively important heterogeneity [56, 57]. For instance, not all of Asch’s or Hardy’s subjects complied with group opinion and there was a great deal of variation in how willing Sherif et al.’s campers coalesced into cohesive and functioning groups. In order to address this possibility we test three factors that have been previously identified as covarying with the average propensity to conform: personality traits, self-esteem, and ideology. The most consistent evidence points towards those who change their opinions as being generally more agreeable, neurotic, and having lower self-esteem [58].

Generating hypotheses regarding the import of other personality and ideological dispositions on opinion change for political, moral and identity-laden topics is more complicated. Extant research indicates support for both stability and change for these traits and differs in the source of that change, i.e., whether it is informational or social. For example, on the one hand we might expect those who are more politically conservative to be more likely to conform to the group overtly, given extant studies showing conservatives think less negatively toward conformity and comply more often to group pressure and norms [59–61]. In addition, conservatives are also higher on the Conscientiousness personality trait, and this trait both reflects and is related to more conformist behavior [62–64].

On the other hand, conservatism, by definition, advocates the status quo and is related to resistance to change and greater refusal to privately accept new information, specifically if that information contradicts one’s values [65, 66], leading to a greater likelihood of internal stability. In a similar manner, those high in openness and more politically liberal, while more likely to take in new information, and thus possibly more likely to privately accept it, are also less prone to restrictive conformity, and thus possibly less likely to conform publicly [67]. We treat these propositions as secondary hypotheses, and explore their import in a limited manner given restrictions in the data.

## Materials and methods

In order to explicate the independent and joint effects of compliance and private acceptance, we designed an experiment, conducted at the Pennsylvania State University from May to December of 2013, which placed participants in a deliberative environment where they faced unified opposition to their expressed opinion on a political topic that is relevant to their local community. We assessed participants’ privately-held opinions, absent the group, before and after the treatment in order to determine whether those who expressed a change in opinion during the discussion only did so verbally in order to comply with the group and gain acceptance or if they privately accepted the group’s opinion and truly updated their own values. The group discussed the topic openly, for approximately 30–45 minutes, also allowing us to assess participant behavior throughout the discussion. We discuss the specifics in more detail below.

In designing the experiment, we leveraged a unique time in Penn State’s history, the aftermath of the Jerry Sandusky child abuse scandal and the firing of longtime Head Coach Joe Paterno. The firing provided an ideal topic of discussion and a hard test of conformity pressure given the fact that it exhibited high salience on campus, was politically charged, and connected to the participants’ identities as Penn State students. The question posed to our participants was whether or not they felt that Coach Paterno should have been fired by the University’s Board of Trustees in November 2011. Previous research demonstrates that undergraduates may not have as clearly defined political attitudes as older adults on many topics and thus may be more susceptible to conformity pressure from peers due to non-attitudes [68]. This informed our choice of the discussion topic, as Paterno’s role in the abuse was not only highly salient on the Penn State campus, but typically invoked strong and diametrically opposed opinions in the undergraduate population and the general Penn state community. We begin by providing some background on this issue and its connection to identity and politics.

### Firing of Penn State football Head Coach Joe Paterno

The first week of November 2011 was a whirlwind for students at Penn State. Police arrested former defensive coach Jerry Sandusky on charges of child sexual abuse following the release of a grand jury report by the Pennsylvania Office of the Attorney General. In the midst of a national media firestorm and with evidence mounting that the University President, Athletic Director and Head football Coach had been aware of Sandusky’s activities, Penn State President Graham Spanier resigned and the Board of Trustees relieved Paterno of his duties. They also placed the Athletic Director, Tim Curley, and Vice President, Gary Schultz, on administrative leave after being indicted for perjury regarding their testimony about their knowledge of Sandusky’s sexual assaults of young boys. Immediately after the firings and suspensions, students poured into campus and downtown State College, causing damage and flipping a news van [69]. Various student protests persisted for weeks. The following summer brought Sandusky’s conviction, but controversy has not subsided, especially in Pennsylvania. The firing is continually alive at Penn State, as lawsuits against the university and the trials of Spanier, Curley, and Shultz continue to progress as Paterno’s family and supporters seek to restore his legacy.

While the real-life context of our design adds to its external validity, the discussion topic’s high salience and likelihood of evoking a strong opinion also improves the internal validity of the experiment. Paterno was more than an employee; he was the image of Penn State, “an extension of [the students’ and alumni’s] collective self” ([70], 154), and thus tied to students’ identities as members of the community [71]. As reported at the time of the scandal:

“More than any other man, Mr. Paterno is Penn State–the man who brought the institution national recognition… Paterno is at the core of the university’s sense of identity.”

[72].

Given the emotion surrounding this issue, it is not unlike morality policies that evoke strong responses from individuals [73], thereby providing a hard test of conformity pressure on value- and identity-laden opinions. There is no better example of this than the ongoing pursuit of justice by the children subjected to abuse by Catholic priests and the mounting evidence of systematic concealment and enablement of such abuse by the Catholic Church. The similarities between Penn State and the Church persist on nearly every level, including the scandals threatening an important aspect of its members’ identities. In this way, the experience of students following the child abuse scandal at Penn State generalizes to politically relevant circumstances where organizational power and personal identities are challenged.

In addition to being a highly salient and identity-laden topic of discussion, the Paterno firing is a social and political issue. It weighed heavily on the 2012 Board of Trustees elections, when many candidates campaigned on their support for Paterno. Furthermore, Pennsylvania Governor Tom Corbett was a de facto member of the Board and originally launched the Sandusky investigation while serving as the state Attorney General. As a board member, Corbett advocated for Paterno’s firing and faced both praise and criticism across the Commonwealth. As a result of the scandal, Pennsylvania passed legislation that clarifies responsibilities for reporting child abuse and heightens penalties for failures to report. The abuse received national recognition. When asked for his reaction to the firing, President Obama called on Americans to search their souls and to take responsibility for protecting children [74]. Thus, there is recognition by elites, the public, the media, and the academy that Paterno’s firing is an inherently political issue. Furthermore, the topic has personal importance to the participants, is identity laden, and relevant at the local, state, and national-levels. Having described the context of the topic of discussion, we now turn to describing the experimental protocol.

### Participant recruitment

The experiment was advertised as a study on political discussion in upper- and lower-level social science courses, as well as through campus fliers and a university research website. As an incentive, participants were entered into a raffle for one of eight $25 gift cards to Amazon. The first participants completed the study in May 2013 and data collection closed in December 2013. There were no major developments in the Sandusky scandal during our data collection phase, thus we believe that no outside events threaten the validity of the study. The firing of the four university officials, Joe Paterno’s death, Jerry Sandusky’s conviction, issuance of the Freeh Report, and the National Collegiate Athletic Association’s sanctions all occurred prior to the start of data collection. This study was approved by the Pennsylvania State University Office for Research Protections Institutional Review Board (Study# 41536) on February 20, 2013. All participants in the treatment group signed a written informed consent form prior to participating in the study. Participants in the control group supplied implied consent by completing the online survey after reading an informed consent document on the first web page of the survey. Penn State’s IRB approved both methods of consent. Consent materials can be found with other study reproduction materials at the corresponding author’s dataverse (http://dx.doi.org/10.7910/DVN/YVCPDT). Thus, all participants provided informed consent and all procedures contributing to this work complied with the ethical standards of the relevant national and institutional committees on human experimentation and with the Helsinki Declaration of 1975.

A total of 58 students participated in either the treatment or control groups. Compared to observational studies, this may appear a small number, but it comports with current research norms that require high participant involvement and a substantial amount of their time [75, 76] and is consistent with the sample sizes for the foundational work in this area [2, 6]. The pre- and post-test, discussion session and debriefing required at least 1.5 hours of each participant’s time. Researchers spent, on average, at least eight hours per participant recruiting, coordinating, and scheduling discussion groups, running discussion sessions, and coding behavioral data. The study generally targeted current undergraduates, but three graduate students and one recent graduate also participated. Upon volunteering to take part in the study, participants were randomly assigned to either the treatment (n = 34) or control (n = 24) group using a coin flip. The total sample includes an un-randomized 16 person pilot of the experimental protocol. See S3 File for additional information on this pilot group, its characteristics, and analyses showing their inclusion does not affect the main findings.

### Pre-test survey

**Fig 1:**
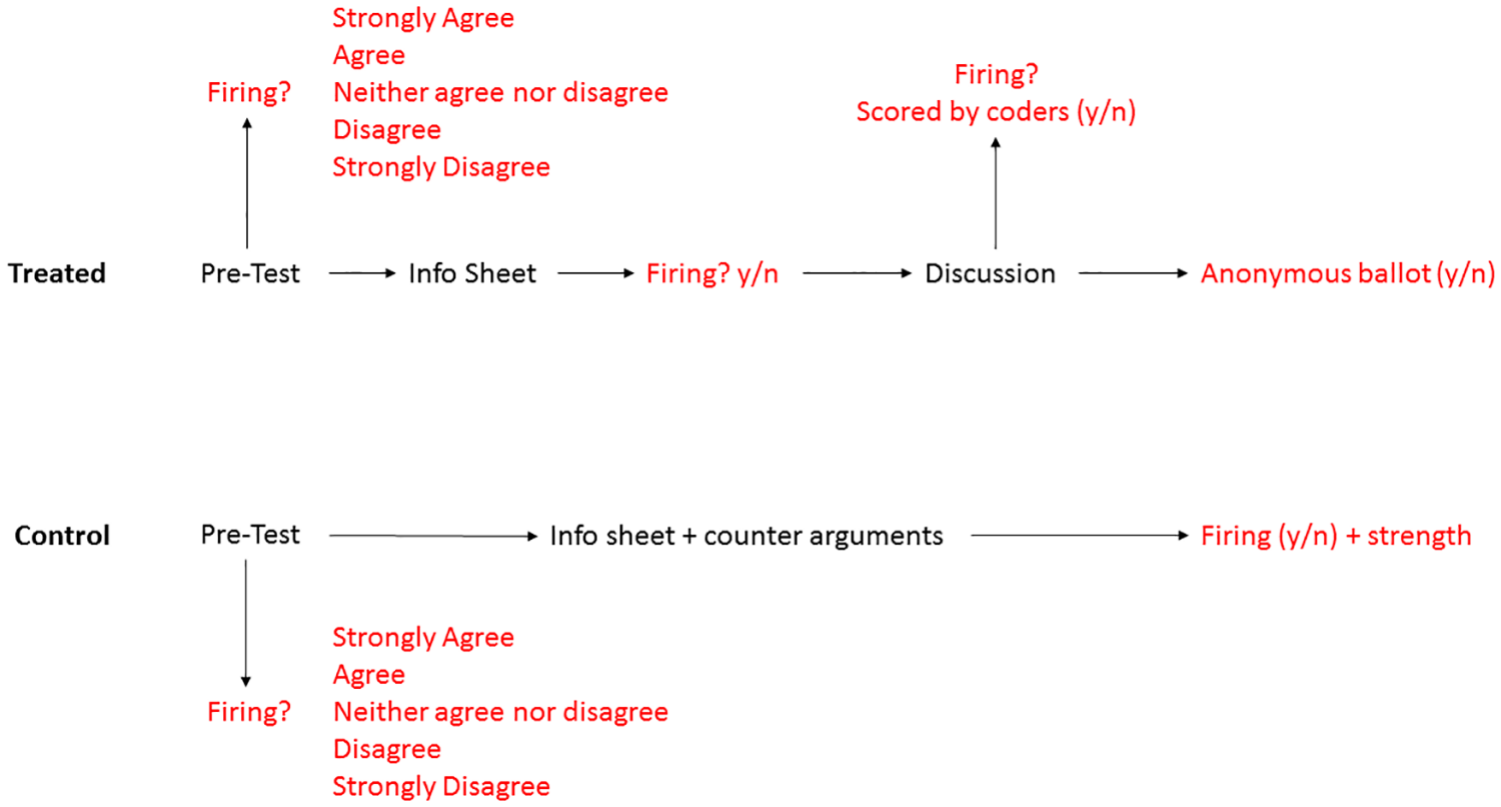
Study design. This figure presents each phase of the study, including information provided to treated and control groups (in black) and the points at which we measured their opinion of the Paterno firing (in red).

Fig 1 presents the study design including information provided to the treatment and control groups (in black) and the points at which we measured their opinion regarding Paterno’s firing (in red). Both groups were administrated a pre-test survey using Qualtrics. The treated group completed this survey before attending a discussion session. In addition to basic demographic characteristics, we collected a number of psychological and behavioral traits for every participant. Ideology was measured by an attitudinal measurement of ideology, a Liberalism-Conservatism scale [77] widely used to prevent measurement error that arises from the difficulty in accurately collapsing a complex view of politics into a single dimension. This measure of ideology is well validated (e.g., Bouchard et al. 2003) and serves as the basis for modern definitions of ideology across disciplines [78, 79]. The measure relies on respondents simply agreeing or disagreeing with a broad range of political and social topics, from evolution to taxes. In this case, we used 48 different topics, which generate an additive scale of conservatism ranging from 0 (very low) to 48 (very high). In addition to measuring our participant’s political ideology, we assessed their self-esteem using Rosenberg’s [80] scale and personality using McCrae and John’s [81] 44-question Big 5 dimensions of personality: openness to experience, conscientiousness, extraversion, agreeableness, and neuroticism.

Finally, all participants were asked their opinion on five policies that affect undergraduates at Penn State: alcohol possession on campus; government oversight of academic performance; the firing of Paterno; prevention of State Patty’s Day celebrations; and use of the student activities fee. Participants recorded their opinion using a five-point Likert scale from “strongly agree” to “strongly disagree.” We included five different topics on the survey so that treatment group participants would be unsure as to which topic they would be discussing.

### Discussion group

**Fig 2:**
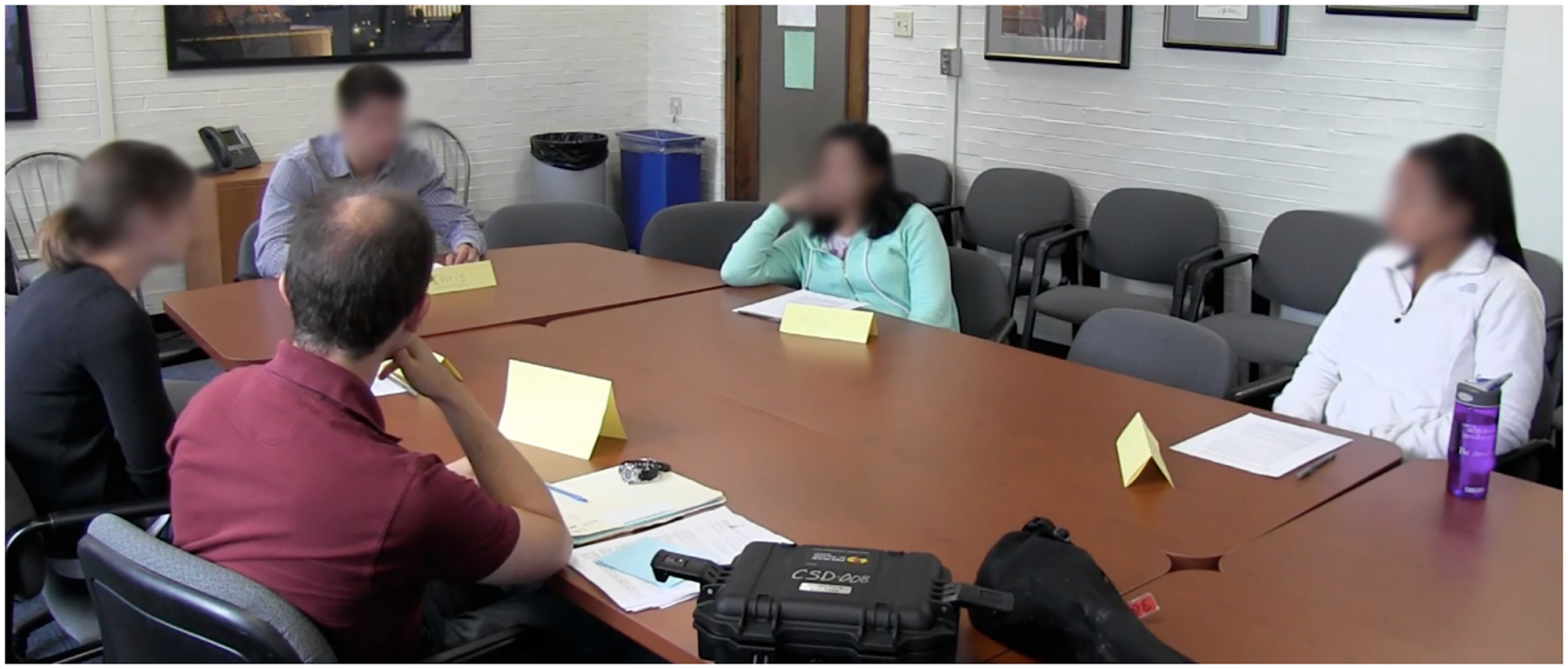
Picture of treatment environment. Clockwise from bottom left: Experimenter, confederate, confederate, participant, and confederate. Note the participant’s seemingly disengaged body language. This participant ultimately changed their opinion.

After completion of the online survey, participants in the treatment group were scheduled individually for a discussion session. Each discussion group was comprised of a single participant and two to four trained confederates (we compare differences in the number of experimenters and find no effects; for more information see S4 File). A total of five unique confederates, three females and two males, were used across the length of the study. Among them were four political science Ph.D. candidates of varying experience and one recent graduate who majored in political science. The confederates looked young and dressed informally, and were not distinguishable from our undergraduate students. In terms of training, the confederates were not strictly scripted so that the discussion would not appear forced or scripted. Instead, the experimenter and other volunteers took part in pre-experiment tests as mock participants so that the confederates could argue both sides of the Paterno firing and develop the consistent points they used for the duration of the study (see S2 File). Fig 2 shows a typical discussion session. Discussion sessions were held in a conference room with all of the group members sitting around a table. There was no fixed seating arrangement.

At the beginning of each discussion session, the experimenter reminded the group that the general purpose of the experiment is to understand political decision-making and how individuals form political opinions. They were told that a topic was randomly selected for each discussion group from the five included in the pre-test survey, with their topic being the firing of Paterno. Prior to the start of open discussion, group members were provided a sheet of excerpts from the Freeh Report [82] regarding Paterno’s involvement in the Sandusky scandal at Penn State (see S1 File). They were told that the information was drawn from independent investigations and was meant to refresh their memories, given that two years had passed since the firing.

After providing time to read the information sheet, the group was polled verbally regarding whether or not they believed Paterno should have been fired (yes or no). The participant was always asked to answer first. This allowed the confederates to subsequently express the opposite opinion throughout the discussion. Though very little time passed between completion of the pre-test surveys and participation in the discussion groups, we did not rely on the opinions expressed in the pre-test surveys as the basis of our confederates’ opinion. We recorded and used the verbal response as the respondents’ opinion. This also ensures that our confederates were responding to the precise opinion held by the participant at the start of the discussion session. This way we could track the effect of conformity pressure on their opinion throughout the session.

The group was then provided 30 minutes for open discussion; however, discussion was allowed to go beyond 30 minutes in order allow participants to finish any thoughts and reflect a more natural interaction. During this discussion, up to four confederates argued the opposition position to greater or lesser degrees depending on the confederate, including responding to and interacting with the participant and even agreeing with the participant on certain points. At the conclusion of the discussion time, group members were told that researchers wished to understand their true opinion at that moment and that we would be aggregating the individual opinions from our groups in order to gain a sense of overall student opinion on each of the five topics. Thus, they were instructed to complete an anonymous ballot with their final opinion. The anonymous ballot allowed us to measure whether their opinion had actually changed during the discussion, conforming to other people’s behavior due to private acceptance that what they are saying is right, or were only publicly complying with other people’s behavior, without necessarily believing in what they are doing or saying.

Each discussion session was video recorded for the purposes of coding both verbal and non-verbal indications of their opinion. Two coders were hired to review each discussion session video and record a series of behavioral characteristics of the participants (not reported in this paper) as well as their impression regarding whether the participants verbally changed their opinion during the course of the discussion (a binary yes/no). The principal investigators also coded each video. We used the modal code from all four coders, with the principal investigators re-reviewing the videos to break six ties. Fleiss’s Kappa [83] indicates moderate agreement among raters on the verbally expressed opinion (0.54, *p* < 0.001).

The combination of anonymous balloting and video recording for verbal cues is an important aspect of the study design that allows us to pull apart whether participants conformed out of a desire to be right, liked, or a combination of the two. Finally, we debriefed each participant to explain the full purpose of the study, including any and all possible points of deception, and to gather information about their personal feelings on being in the minority during the discussion.

### Control group

We utilized a control group in order to identify the independent effect of social pressure on opinion change. Their behavior established a baseline expectation for the amount of opinion change we could expect with just the introduction of new information and no interpersonal interaction. This baseline then allows us to compare the two groups, social influence treatment and control, in order to tease apart the independent and joint effects of social conformity pressure and information on opinion change.

To this end, the control group took the same pre-test survey as the treatment group. However, after completion of the survey, instead of being in a deliberative session, control group participants read additional information on a topic that was “randomly” selected from the five opinion questions. Based on their opinion regarding the firing of Paterno, we presented them with the same sheet of information provided to the treated as well as a summary of the same pro- and counter-arguments used by the actual confederates during the discussion group sessions (see S1 and S2 Files). After reading these, control group participants were asked whether they believe Paterno should have been fired (yes or no) and the strength of that opinion (very strongly, somewhat strongly, neutral). If they changed their opinion at this juncture, we consider they did so only because of the introduction of new information, as there was an absence of social pressure. Thus, our design allows us to parse out the effect of the discussion group and the social pressure emerging from an unanimity of opinion opposite to the participants.

## Results and discussion

**Fig 3:**
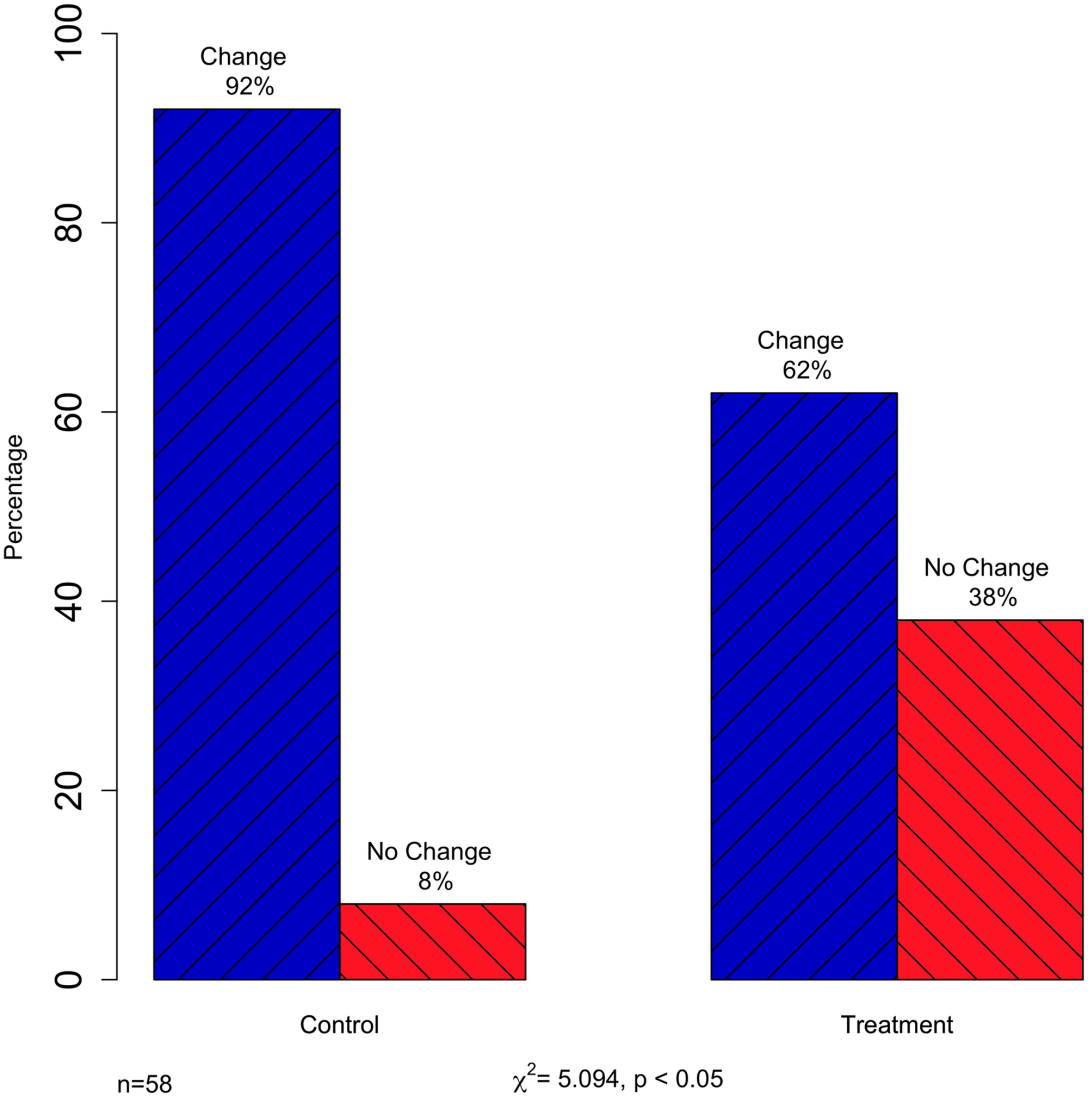
Discrete change of opinion in control and treatment groups.

The core finding of this study revolves around the question to what extent will people conform to an opposing opinion on a topic that is salient, politically charged, and informs some aspect of their identity? Furthermore, can we evoke deviation rates similar to the foundational studies that relied on less complex aspects of one’s psychology [1]? And most important, what type of change is occurring? For those participants who changed their opinions, was it due to new information (i.e., private acceptance), social pressure (i.e., public compliance), or some combination of the two? To answer these questions, we first examined the degree of opinion change in both the treatment and control groups. For the control group, we compared their initial opinion from the pre-test survey with the opinion they provided after reading the information sheet and counter-arguments. Fig 3 displays the percentage of each group that did and did not change their opinion. Within the control group, which received the same information as the discussion group, but had no social interaction, only 8 percent of the participants changed their opinion. The information-based change we observed is consistent with extant research [84, 85]. In addition, though a large proportion of the control group did not change their opinion, some did moderate it (i.e., strengthened or weakened) based on the receipt of new information alone. See S5 File for a further breakdown of these changes.

Turning to the treatment group, 38 percent of our treated participants changed their opinion between the initial vote (after receiving information and prior to the discussion) and the final secret ballot. Our complex, identity, and value-laden topic returned findings that comport remarkably close to the deviation rates of Asch [2] and those that follow (for a meta-analysis, see [6]). If we consider all other things equal, the 30 percent increase in opinion change is dependent on the treatment of participating in the group discussion (χ^2^ = 5.094, *p* < 0.05). This finding remains unchanged if the 16 non-randomized members of the pilot study are removed from the treatment group (though the p-value of the chi-square declines to 0.10, due to the smaller n, see S3 File). As further evidence, Table 1 presents logistic regression results demonstrating the treatment effect. Namely, being in the treatment condition increases the odds of opinion change by 581 percent. Meaning, social pressure and/or the personal delivery of information, as opposed to simple exposure to new information, had a profound influence on either true opinion change through private acceptance or conformity through public compliance. Due to the small sample size, we are hesitant to include additional covariates in this model, but instead use t-tests below to examine differences in the characteristics of participants who changed their opinion and those who did not.

**Table 1:** Effect of treatment on opinion change.

Variable	Coefficient	Odds Ratio
Treatment	1.92*	6.81
	(0.74)	[1.63, 47.00]
Intercept	-2.40*	
	(0.74)	

N = 58;

* *p* < 0.05; standard errors in parentheses and 95% confidence intervals in brackets

### Sources of change

Moving to our secondary analyses, the research design also allowed us to parse out the specific sources of change within the treatment group. Recall we accounted for both true opinion change (i.e., the anonymous ballot at the end of discussion) and verbal opinion change (i.e., declared opinion change during group discussion captured in video and coded by independent raters) for those in the treatment condition. Therefore, we divided those in the treatment group into four subgroups in order to better understand why they changed their opinion. Table 2 shows the percentages of participants in the treatment group who changed their opinion overtly, covertly, or not at all. In sum, 47 percent did not change their opinion between the start and end of the discussion session. A total of 33 percent changed both overtly and covertly, meaning they verbally expressed an opinion change and wrote a changed opinion on their secret ballot. We argue that this group responded to a combination of the desires to be right and liked. Of the remaining participants, 10 percent changed due to a desire to be liked (overtly, but not covertly) and 10 percent due to a desire to be right (covertly, but not overtly). Though only anecdotal, one of the participants in the desire to be right category went so far as to tell the experimenter that he agreed with the group but adamantly refused to agree openly. Such participants were swayed by the introduction of new information out of a strong desire to be right, but apparently did not want to look like they were changing their opinion. Thus, our first set of analyses confirms that information plays an important role in opinion change, but social pressure also has a substantive and, at least in this context, a larger effect. For even a topic so important to one’s identity, participants changed their previously held opinions.

**Table 2:** Percentage of treatment group participants that changed their opinion either overtly or covertly, both, or neither.

	**Covert Change**	**No Covert Change**
**Overt Change**	33 percent	10 percent
**No Overt Change**	10 percent	47 percent

N = 34, only includes treatment group

### Psychological differences

Having established the main findings of our study and the relative import of the two causal mechanisms for why participants changed their opinion, we now turn to examining how underlying traits, including ideology, personality, age and sex, differ between those that changed their opinion and those that did not. Demographic differences are included for descriptive purposes. First, we assessed differences between pro- and anti-firing participants. Second, we examined the relationship between direction of opinion change and trait differences between participants that changed their opinion and those that held firm. Due the nature of the experiment and specific focus on the question of causality, these tests are secondary to the main findings in the paper. For the following analyses, the sample sizes are small and in some cases and the findings only speculative.

**Fig 4:**
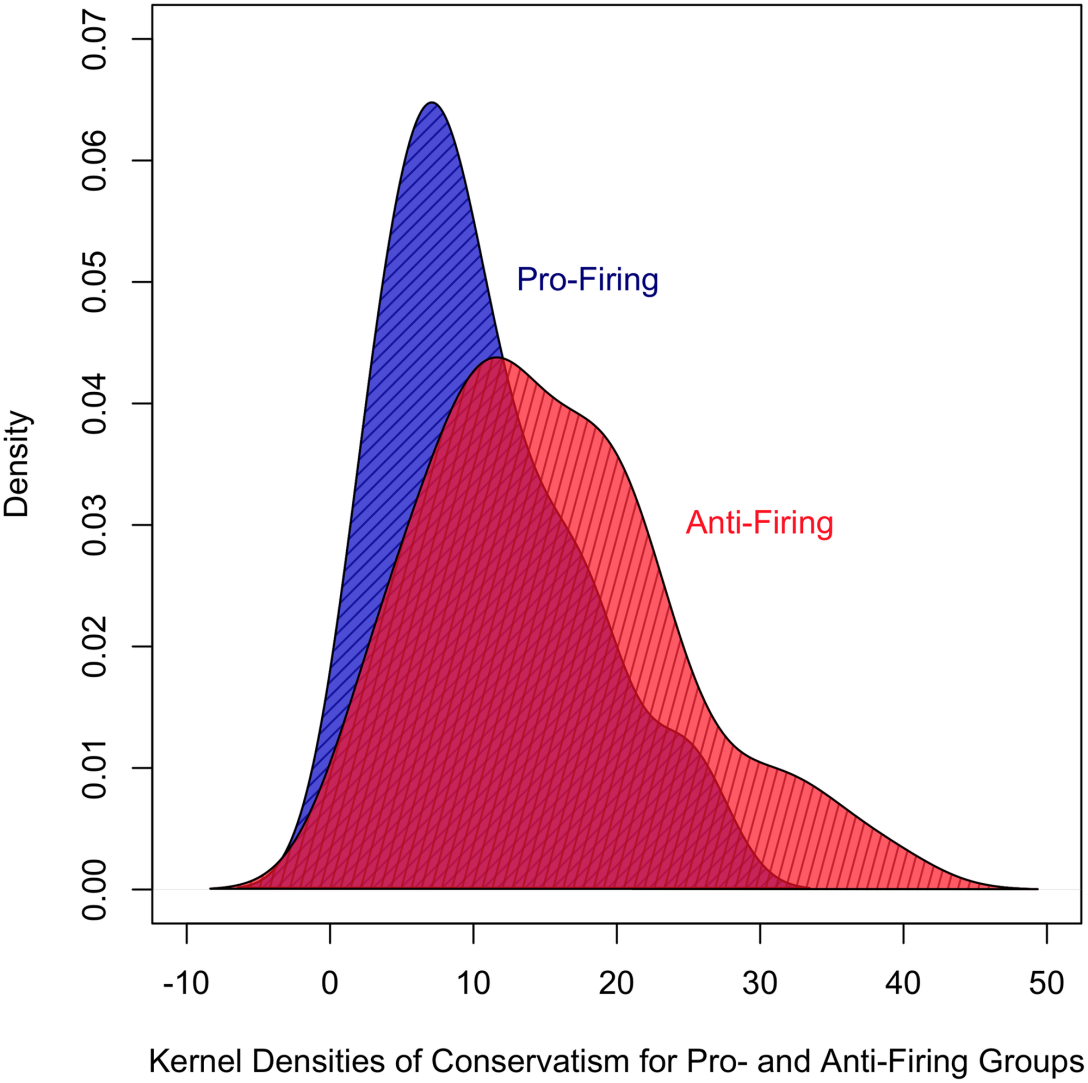
Distribution of conservatism for pro- and anti-firing groups of participants.

Across both the treatment and control groups, the pre-test survey showed almost two-to-one support for Paterno keeping his job (i.e., against the firing). As mentioned earlier, “JoePa” was not only a symbol of Penn State, but also an icon to its students, and to some degree seen as a reflection of them. Table 3 displays the average demographic and psychological measures for those for and against the firing, based on the pre-test survey. The only statistically significant difference between the groups is their political ideology. The group opposed to Paterno’s firing is, on average, more conservative in their attitude positions than those that called for his firing. It is important to note that these are college students, and thus the overall distribution of ideology exhibits a liberal skew. However, Fig 4 demonstrates that the pro-firing group is not only less conservative, on average, but is also more ideologically narrow, whereas those that did not support the firing are more conservative, but also drawn from a wider ideological span. This finding suggests that ideology is a substantial factor for individuals that supported the firing. Whereas support for Paterno may have a less pronounced ideological dimension, those supporting his firing may focus more narrowly on the issue of child abuse and the responsibility of those in leadership to protect vulnerable citizens. Given that ideology is the only difference we could identify among participants’ opinions prior to the start of the experiment, we next examined whether there were differences between participants who changed their opinion and those that did not in both the treatment and control conditions.

**Table 3:** Comparison of participants who indicated support or opposition for the firing of Paterno in their pre-test survey, including t-tests.

Variable	Pro-Firing Mean	Anti-Firing Mean	Difference
	(St. Dev.)	(St. Dev.)	[95% Conf. Int.]
Age	22.85	22.10	0.75
	(4.71)	(2.02)	[-2.16, 3.66]
Male^†^	0.54	0.55	-0.01
	(0.53)	(0.51)	[-0.34, 0.32]
School Year	3.46	3.45	0.01
	(1.27)	(1.34)	[-0.87, 0.90]
Conservatism	10.54	16.42	-5.88*
	(6.68)	(9.06)	[-10.92, -0.85]
Self-Esteem	31.38	28.61	2.77
	(6.05)	(6.46)	[-1.44, 6.98]
Extraversion	18.85	20.94	-2.09
	(6.40)	(5.62)	[-6.35, 2.17]
Agreeableness	26.62	24.90	1.72
	(5.81)	(5.62)	[-2.17, 5.60]
Conscientiousness	26.00	25.71	0.29
	(4.18)	(3.76)	[-2.51, 3.09]
Neuroticism	12.77	12.13	0.64
	(6.48)	(6.27)	[-3.76, 5.04]
Openness	28.08	26.97	1.11
	(2.63)	(3.83)	[-0.93, 3.15]
Observations	13	31	

* entries indicate significant t-tests, p < 0.05.

^†^Difference in proportions test used for Male. These analyses have a smaller overall sample size due to removal of neutral pre-test votes.

Tables 4 and 5 provide a sense of how demographic and psychological characteristics differ between participants who changed their opinion and those who did not. Table 4 includes both treatment and control participants, whereas Table 5 focuses solely on the treatment group. We found evidence both supporting and refuting our hypotheses presented above. There were consistent significant differences (*p* < 0.05) in conservatism and conscientiousness. Namely, participants who changed their opinion are less conservative and less conscientious. Given the reported relationships between these two traits, this finding makes sense. Additionally, when all subjects are pooled (Table 4), there is also a significant difference in neuroticism, with opinion changers registering higher on this scale. Both suggest that political and psychological traits may play a role in the mean shift demonstrated above. There were no differences based on the number of confederates. Meaning, participants were no more or less effected by social pressures from greater (4) or fewer (2) opponents in the discussion environment. These results demonstrate that individual differences exist across individuals that change their opinion and those that do not. Additional research will be required to both confirm and expand upon these findings. What we do find, however, is in line with expectations derived from past research and points to useful areas of future inquiry.

**Table 4:** Comparison of participants in both treatment and control conditions who changed their opinion, including t-tests.

Variable	Change Mean	No Change Mean	Difference
	(St. Dev.)	(St. Dev.)	[95% Conf. Int.]
Age	21.80	22.69	-0.89
	(2.14)	(3.39)	[-2.43, 0.65]
Male^†^	0.46	0.60	-0.14
			[-0.42, 0.15]
School Year	3.27	3.60	-0.33
	(1.75)	(1.33)	[-1.37, 0.70]
Confederate Total	3.46	3.38	0.08
	(0.66)	(0.59)	[-0.38, 0.54]
Conservatism	9.80	16.00	-6.20*
	(5.28)	(8.93)	[-10.09, -2.31]
Self-Esteem	27.33	29.63	-2.30
	(5.38)	(6.64)	[-5.81, 1.22]
Extraversion	19.87	21.28	-1.41
	(3.96)	(6.22)	[-4.24, 1.41]
Agreeableness	27.07	25.70	1.37
	(4.10)	(5.60)	[-1.39, 4.13]
Conscientiousness	24.07	26.33	-2.26*
	(3.47)	(3.87)	[-4.46, -0.06]
Neuroticism	15.73	11.77	3.96*
	(6.60)	(5.49)	[0.02, 7.91]
Openness	28.13	26.93	1.20
	(3.25)	(3.63)	[-0.86, 3.26]
Observations	15	43	

* entries indicate significant t-tests, p < 0.05.

^†^Difference in proportions test used for Male.

**Table 5:** Comparison of participants in only the treatment condition who changed their opinion, including t-tests.

Variable	Change Mean	No Change Mean	Difference
	(St. Dev.)	(St. Dev.)	[95% Conf. Int.]
Age	21.69	23.29	-1.60
	(2.29)	(4.30)	[-3.90, 0.72]
Male^†^	0.48	0.62	-0.14
			[-0.48, 0.20]
School Year	3.08	3.81	-0.73
	(1.80)	(1.33)	[-1.94, 0.47]
Confederate Total	3.46	3.38	0.08
	(0.66)	(0.59)	[-0.38, 0.54]]
Conservatism	8.85	15.33	-6.48*
	(4.86)	(10.78)	[12.03, -0.95]
Self-Esteem	27.08	28.81	-1.73
	(5.57)	(7.59)	[-6.42, 2.96]
Extraversion	19.69	21.14	-1.45
	(4.23)	(5.37)	[4.84, 1.94]
Agreeableness	26.77	26.05	0.72
	(3.83)	(5.60)	[-2.58, 4.02]
Conscientiousness	23.92	27.86	-3.94*
	(3.73)	(3.75)	[-6.65, -1.22]
Neuroticism	15.15	11.86	3.29
	(6.93)	(5.34)	[-1.38, 7.97]
Openness	28.46	26.57	1.89
	(3.15)	(6.69)	[-1.58, 5.36]
Observations	13	21	

* entries indicate significant t-tests, p < 0.05.

^†^Difference in proportions test used for Male. Smaller overall sample size due to using only treatment condition participants.

### Debriefing

All participants were debriefed upon completion of the discussion and informed to all aspects of the study. Participants were asked during the debriefing how they felt about being the only dissenting voice. Forty-seven percent of the treatment group participants freely offered that they felt pressured or intimidated. Twenty-nine percent also freely said that they felt like they had to dig in and defend their position during the discussion. This included six people that ultimately changed their minds. One said, “I’m not getting any support in this room. Alright I’ll defend my own position.” Another said, “I feel extra pressure to explain myself.” For some, their defensiveness continued into the debriefing. In particular, some students that did not change their opinion continued defending themselves when talking one-on-one with the experimenter, even after it was explained no matter which position they took, they would face opposition. This demonstrates that some participants are put on the defensive when faced with a unified opposition. Of those that expressed feeling defensive, some dug-in deeply and did not budge at all, while others opened up to the influence of their peers as the discussion progressed. This behavior comports the foundational work of Asch [1, 2] and Milgram [86] and strongly suggests that our participants indeed experienced social pressure in the treatment condition, but differs in that it highlights the variance in how individual’s react to such pressure.

## Limitations

We wish to call attention to two specific limitations of this study that are discussed above and in the supplementary materials, but warrant further mention. The first limitation is the inclusion of a meaningful, relative to the overall sample size, non-randomized pilot of the treatment condition. While this had no substantive effect on the results, it is important to recognize and we discuss this in more detail in the S3 File. Second, Fig 1 makes apparent that we use two similar, but slightly different scales for opinion throughout the study. Namely, pre-test opinion is measured on a five-point Likert scale and the remaining opinion measures are dichotomous (yes/no), with an additional strength question for the control group. Our primary analyses, however, rely on the comparison of the two yes/no answers in the treatment group; the verbal designation of yes/no at the beginning of the discussion section and the yes/no in the post discussion ballot. We further discuss this in the S5 File.

Finally, to some the small sample size of the study may be a limitation, especially those concerned about a replication crisis in Social Psychology [87]. We would respond, however, that the intensive nature of this study in terms of researcher hours and treatment condition makes it difficult to scale-up. Thus, a multi-site replication is likely the best approach to assessing the veracity of these findings [88, 89]. We encourage such replication and have provided all materials necessary on the corresponding author’s Dataverse (http://dx.doi.org/10.7910/DVN/YVCPDT). Additional lessons relevant to replication work and laboratory experiments in political science can be found in Mallinson (2018) [90].

## Conclusions

While researchers have examined the roles of social influence (public compliance) and new information (private acceptance) on opinion change, the two are less often examined concurrently and the explicit causal arrows are more often assumed than tested through an experiment. Furthermore, social conformity is a complex concept to measure through surveys or interviews alone. Live interaction provides an optimal means to understand social pressures. Our experiment was designed specifically to further unpack the causal mechanisms underlying opinion change and test whether a person’s values and identity are subject to social pressure. Furthermore, the selection of the topic of study, the firing of an important symbol of Penn State, also allowed us to explicate the extent to which information and social pressure challenge a person’s deeply held values and identity. We find that while information has an important role in changing people’s opinions on a highly salient topic that is attached to a group identity, the social delivery of that information plays a large and independent role. Most individuals that changed their opinion did so out of some combination of the two forces, but there were people who only changed their opinion overtly in order to gain social acceptance as well as those who did not want to give the appearance of changing their mind, but still wanted to be right.

These findings have important implications for research on social and political behavior. They reinforce the understanding that citizens and elites cannot be simply viewed as rational utility maximizers independent of group dynamics. Yet, at the same time, the desire to be right and information remain critical components of opinion change. Furthermore, there are important individual differences such as ideology, self-esteem, and personality that appear to have a role in conformity. Exposure to politics and political discussion are fundamentally social, and therefore behavior is conditioned on the combination of the information one receives, and the social influence of the person or group providing that information interacting with one’s disposition. All should be considered when examining any inter-personal, social or political outcome. Be it a deliberative setting like a jury or a town hall meeting or informal gatherings of citizens, or political elites for that matter, changes in behavior are not simply due to rational information-driven updating, and even when they are, that updating may be pushed by the social forces that we experience in our interactions with other humans in variegated ways dependent upon the characteristics of the individual (for example, see [91]). This was the case for simple and objective stimuli, like Asch’s lines, and it is also the case in our context-laden experiment that focuses on the complexities of personal identity and opinion. That is, the conformity of social and political values relies on the same psychological mechanisms underlying general conformity.

Beyond theoretical and empirical importance for the study of social and political behavior, these findings also hold normative importance for democratic society. The normative implications are perhaps best exemplified by the organizational and personal turmoil that followed the revelation of child abuse by priests in the Catholic Church. Politics forms important aspects of the social and personal identities of elites and citizens, more so today than ever before [92, 93]. People include their political party, positions on particular issues (e.g., environmentalism), and membership in political, religious, social and academic organizations, among other things, as key aspects of their identities. Our experiment helps us better understand how individuals behave when part of that identity is challenged.

That being said, no design is perfect, and this experiment only unpacks part of the causal mechanism. Like the early work on social conformity, it serves as a foundation for future studies to extend upon and further explicate the causal mechanism. For example, an extension on this design, such as controlling variation in the type and number of confederates [44, 94], could help us better understand the nature and amount of pressure necessary to induce conformity across a variety of individual characteristics. For example, a potentially fruitful avenue of extension would be to provide the participant with one supportive confederate who verbally changes their opinion during the discussion. Having support reduces conformity pressure, but deviation by that support should increase it. Additionally, while we identify individuals whose behavior was prompted by either social pressure or information, the largest group responded to a combination of the two. Further parsing out the interaction between information, persuasion, pressure and the complexity of human dynamics will require an even more complex research design on a larger scale. The numerous extensions of Asch’s original experiment demonstrate the wealth of potential extensions of this design that can help unpack this black box. Doing so requires an incremental approach that will be time and resource intensive. This study provides the foundation for those next steps.

## Supporting information

S1 File Information sheet provided to both treatment and control groups. (DOCX)

S2 File Confederate talking points. (DOCX)

S3 File Randomization. (DOCX)

S4 File Use of deception in the study design. (DOCX)

S5 File Breakdown of opinion change in the treatment and control groups. (DOCX)
