# Correction: The effects of information and social conformity on opinion change

**DOI:** 10.1371/journal.pone.0230728

**Published:** 2020-03-18

**Authors:** Daniel J. Mallinson, Peter K. Hatemi

In Fig 3, there is an error in the labeling of the change of opinion in control and treatment groups. The "Change" and "No Change" labels are swapped. In the control group, the percentage of participants that did not change their opinion should be 92%, while the percentage that did change their opinion should be 8%. In the treatment group, the percentage of participants that did not change their opinion should be 62%, while the percentage that did change their opinion should be 38%. The authors have provided a corrected Fig 3 here.

**Fig 3:**
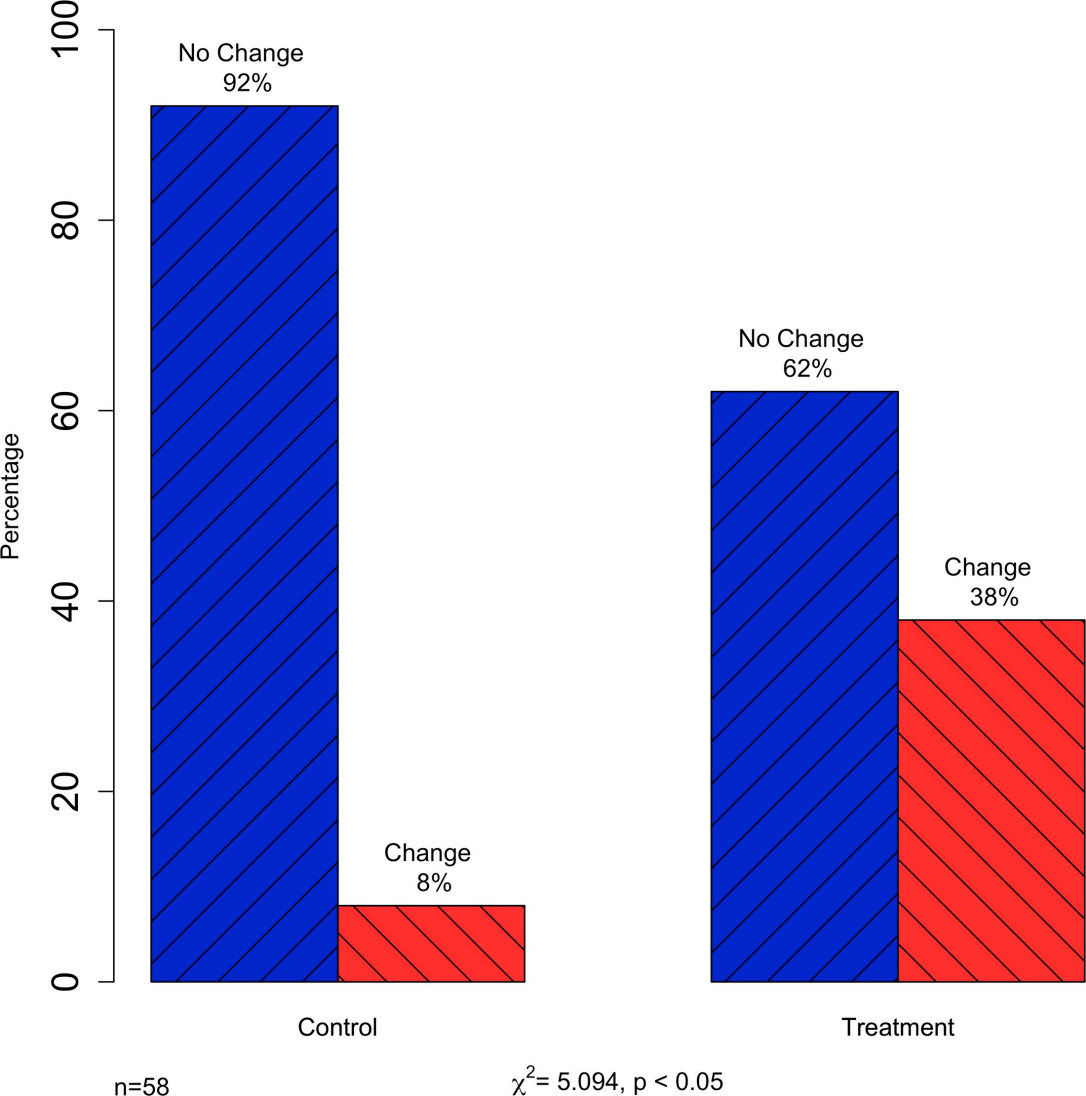
Discrete change of opinion in control and treatment groups.
