# Supplementary material for: The effects of information and social conformity on opinion change
Source: PLoS One. 2018 May 2;13(5):e0196600. doi: 10.1371/journal.pone.0196600 (PMC5931497; doi:10.1371/journal.pone.0196600)
Supplement: S1 File — (DOCX) [file pone.0196600.s001.docx]

**S1 Appendix. Information Sheet Provided to Both Treatment and Control Groups**

Below are summaries and excerpts from an investigation into the Jerry Sandusky matter at Penn State. The information addresses Head Coach Joe Paterno’s knowledge and behavior.

- Police first investigated Sandusky in 1998 for allegedly showering with an 11-year old in the Lasch Building on campus. In May of that year, Athletic Director Curly informed Vice President Shultz and President Spanier that he had “touched base with” Paterno about the incident. Days later, Curley emails Shultz: “Anything new in this department? Coach is anxious to know where it stands.”
- Witnesses interviewed in the investigation reported that Coach Paterno knew “everything that was going on” when it came to the football program and had the authority to control access to the football facilities.
- There is no evidence that Penn State officials, including Coach Paterno, actively interfered with the investigation, but they were kept informed during it. The local district attorney decided not to pursue charges against Sandusky at the conclusion of the investigation. No action was taken to limit Sandusky’s access to university facilities.
- A proposal written in 1999 by Sandusky to continue using the football facilities included a handwritten note from Paterno reading, “Is this for personal use or 2nd Mile kids. No to 2nd Mile. Liability problems.”
- In February 2001, graduate assistant Mike McQueary reported to Coach Paterno that he saw Sandusky behaving suspiciously in the Lasch showers with a young boy. Paterno told him “you did what you had to do. It’s my job now to figure out what we want to do.”
- Paterno reported the incident to Curley and Shultz the following day. Paterno indicated that he delayed because he did not “want to interfere with their weekends.”
- Additional e-mail correspondence between Curley and Shultz indicates that they consulted with Coach Paterno regarding the Sandusky situation in 2001. Subsequent to these discussions, leadership determined that Sandusky should be asked to not bring minors to campus facilities any longer. The incident was not reported to the Board of Trustees.
- The relationship between the University, Sandusky, and the Second Mile charity continued until a grand jury investigation was held in 2011 after the Pennsylvania Attorney General’s Office investigated allegations of Sandusky’s behavior. Paterno, Shultz, Curley, and Spanier are all subpoenaed to testify before the grand jury.
- Paterno consistently testified that he had no knowledge of the 1998 investigation into Sandusky. He said that 2001 was the first incident that he was made aware of.
- In a Washington Post report, Paterno was asked why he had not pursued the 2001 incident further. Paterno responded, “I didn’t know exactly how to handle it and I was afraid to do something that might jeopardize what the University procedure was. So I backed away and turned it over to some other people, people I thought would have a little more expertise than I did. It didn’t work out that way. In hindsight, I wish I had done more.”
