# Supplementary material for: The effects of information and social conformity on opinion change
Source: PLoS One. 2018 May 2;13(5):e0196600. doi: 10.1371/journal.pone.0196600 (PMC5931497; doi:10.1371/journal.pone.0196600)
Supplement: S2 File — (DOCX) [file pone.0196600.s002.docx]

**Supplement S2a. Confederate talking points from the discussion sessions that were provided to members of the control group: Pro-Firing**

Yes, Coach Paterno should have been fired:

- In serious cases (e.g., allegations of rape), someone can be held accountable for not reporting the issue to the police
- Paterno had this authority and responsibility as the head of the football program
- There's enough information to suggest he knew enough about the Sandusky situation that he should have taken the allegations of child abuse more seriously and should have done more to ensure the allegations were being handled by those above him
- It is clear from the independent investigations that he knew of both the 1998 investigation and the 2001 incident in the Lasch Building
- Seems irresponsible that Paterno would privilege the weekends of Curly and Schultz more than the seriousness of the allegations that were brought before him
- There is no evidence that he followed up on the allegations against Sandusky after passing the information higher
- He did not know or follow University procedure, and that is a reasonable grounds for firing him
- He made a mistake and, therefore, should have been held accountable
- Employees are often fired for far less serious reasons (e.g., being late to work)
- He is not held to the standards of a court of law; given the evidence, it is within the right of the Board of Trustees to fire him
- Collegiate coaching is a competitive job and there are plenty of other coaches who could have made a better decision
- In light of heavy public scrutiny, the Board need to take swift action

**Supplement S2b. Confederate talking points from the discussion sessions that were provided to members of the control group: Anti-Firing**

No, Coach Paterno should not have been fired:

- It is easy to sit here with all of the information in front of us more than a decade later and say "Yes, Paterno should have done more," but Paterno was given bit and pieces of information over a long period of time that may have been difficult for him to connect the dots as the situation with Sandusky was unfolding
- Not all the evidence was known; Sandusky had not even been convicted at the time
- The university bureaucracy itself was broken, and it is not fair that Paterno should take the blame for acting appropriately within a system that tells its employees to report issues to higher authorities and do nothing else
- He did what was required; he reported the incident to his superiors
- He responded appropriately each time given the information available to him
- Should be able to trust that the people above you will handle the allegations once they are made aware of them
- In cases of “due obedience,” soldiers are not held accountable for actions/decisions made by their superiors
- Given that the district attorney found no reason to pursue the 1998 investigation of Sandusky, Paterno may have dismissed any reasons to feel Sandusky was behaving inappropriately
- Paterno did tell Sandusky not to bring Second Mile youth onto campus facilities and cannot possibly spend his time making sure Sandusky was not in the showers with them
- It is unfair to fire Paterno for something that was really Sandusky's fault
- The firing of Paterno distracted from Sandusky and from the process of addressing the issue of child abuse
- It is unclear that someone different would have "done more" than what Paterno did
- He was going to retire anyway; so he should have been allowed to step down
- There are many more preferable outcomes to the scandal than the firing of Paterno
- Finger-pointing and name-calling are not a good way to heal from the scandal and move forward as a community
- He was obviously a good man; he made numerous contributions to the community and school which should not be overshadowed by a single bad decision
- We should have given him the opportunity to use his iconic status to make a difference (take a stand against child abuse, lead an investigation into the shortcomings of university procedure, ask him what conditions would have to be different to get him to have called the police, etc.)
