# Supplementary material for: The effects of information and social conformity on opinion change
Source: PLoS One. 2018 May 2;13(5):e0196600. doi: 10.1371/journal.pone.0196600 (PMC5931497; doi:10.1371/journal.pone.0196600)

**S3 Appendix: Randomization**

The data analysis in the main text includes a 16 person pilot of the experimental protocol. This pilot group was used to test the protocol, ensure consistency among the confederates’ talking points, and incorporate the arguments made by the participants into both the confederates’ talking points and the control group survey. Given the time intensity of this study for participants, confederates, and the investigators, we chose to ensure that the protocol was working before implementing the full study ([Thabane et al., 2010](#_ENREF_4)). This ensured that the control group was given the same information as the treatment group, thus better isolating the treatment effect of social pressure. We also conducted a series of mock discussions with the principal investigators and the confederates before conducting the pilot. Thus, we found that our confederates were well prepared with their discussion points even prior to the pilot and in post-experiment analysis of the video recorded sessions we observed that the treatment did not differ substantially between the pilot participations and those subsequently randomized into treatment and control. The confederates were generally consistent across all participants in their specific arguments, either for or against the Paterno firing. Given that the sampling frame and treatment condition methodology were the same, we included the pilot data in our final analysis ([Thabane et al., 2010](#_ENREF_4)).

Given that 16 of the 34 treatment group participants were not assigned randomly, we wanted to test for, and report, any significant differences in demographics and dispositions for the two sub-groups. Table S1 presents these results and there are two significant differences. First, the non-randomized subgroup includes more males than the randomized subgroup. Second, the non-randomized subgroup is, on average, less neurotic than the randomized group.

| **Table S3: Comparison of demographic and dispositional traits for non-randomized and randomized subgroups with the treatment group**   \| Variable \| Non-randomized Mean  (St. Dev.) \| Randomized Mean  (St. Dev.) \| Difference  [95% Conf. Int.] \| \| --- \| --- \| --- \| --- \| \| Age \| 23.50  (5.15) \| 21.94  (1.43) \| 1.56  [-1.25, 4.36] \| \| Male^†^ \| 0.69  (0.48) \| 0.28  (0.46) \| 0.41**  [0.10, 0.72] \| \| School Year \| 3.62  (1.89) \| 3.44  (1.20) \| 0.18  [-0.96, 1.32] \| \| Conservatism \| 14.44  (7.37) \| 11.44  (11.00) \| 3.00  [-3.50, 9.49] \| \| Self-Esteem \| 29.62  (6.46) \| 26.83  (7.20) \| 2.79  [-1.98, 7.56] \| \| Extraversion \| 20.56  (5.60) \| 18.72  (4.27) \| 1.84  [-1.69, 5.37] \| \| Agreeableness \| 26.94  (4.77) \| 25.78  (5.17) \| 1.16  [-2.31, 4.63] \| \| Conscientiousness \| 26.44  (3.95) \| 26.28  (4.46) \| 0.16  [-2.78, 3.10] \| \| Neuroticism \| 10.69  (5.56) \| 15.28  (5.90) \| -4.59**  [-8.60, -0.59] \| \| Openness \| 28.50  (4.00) \| 27.44  (2.97) \| 1.06  [-1.5, 3.56] \| \| Pro-firing^†^ \| 0.19  (0.40) \| 0.28  (0.46) \| -0.09  [-0.51, 0.26] \| \| Observations \| 16 \| 18 \|  \| \| ** entries indicate significant t-tests, p < 0.05, * if using a p < 0.10 benchmark  ^†^Difference in proportions test used for Male and Pro-Firing \| \| \| \| |
| --- | --- | --- | --- | --- | --- | --- | --- | --- | --- | --- | --- | --- | --- | --- | --- | --- | --- | --- | --- | --- | --- | --- | --- | --- | --- | --- | --- | --- | --- | --- | --- | --- | --- | --- | --- | --- | --- | --- | --- | --- | --- | --- | --- | --- | --- | --- | --- | --- | --- | --- | --- | --- | --- | --- | --- | --- |

We also examined whether the main result of the analysis, defection among treated and control participants, changed when the pilot data were removed. Fig A replicates Fig 3 in the main text, but with the pilot subjects in the treatment group removed. The results remain largely the same. The total treatment group participants that did not change their opinion is slightly larger and the group that changed their opinion is slightly smaller (a five percent difference). These changes, combined with the smaller n, does reduce the p-value for the chi-square test to 0.10. Thus, we are reasonably confident that our findings hold even when pilot data are excluded from the analysis of the main effect.

Fig A. Discrete change of opinion in control and treatment groups, excluding pilot participants


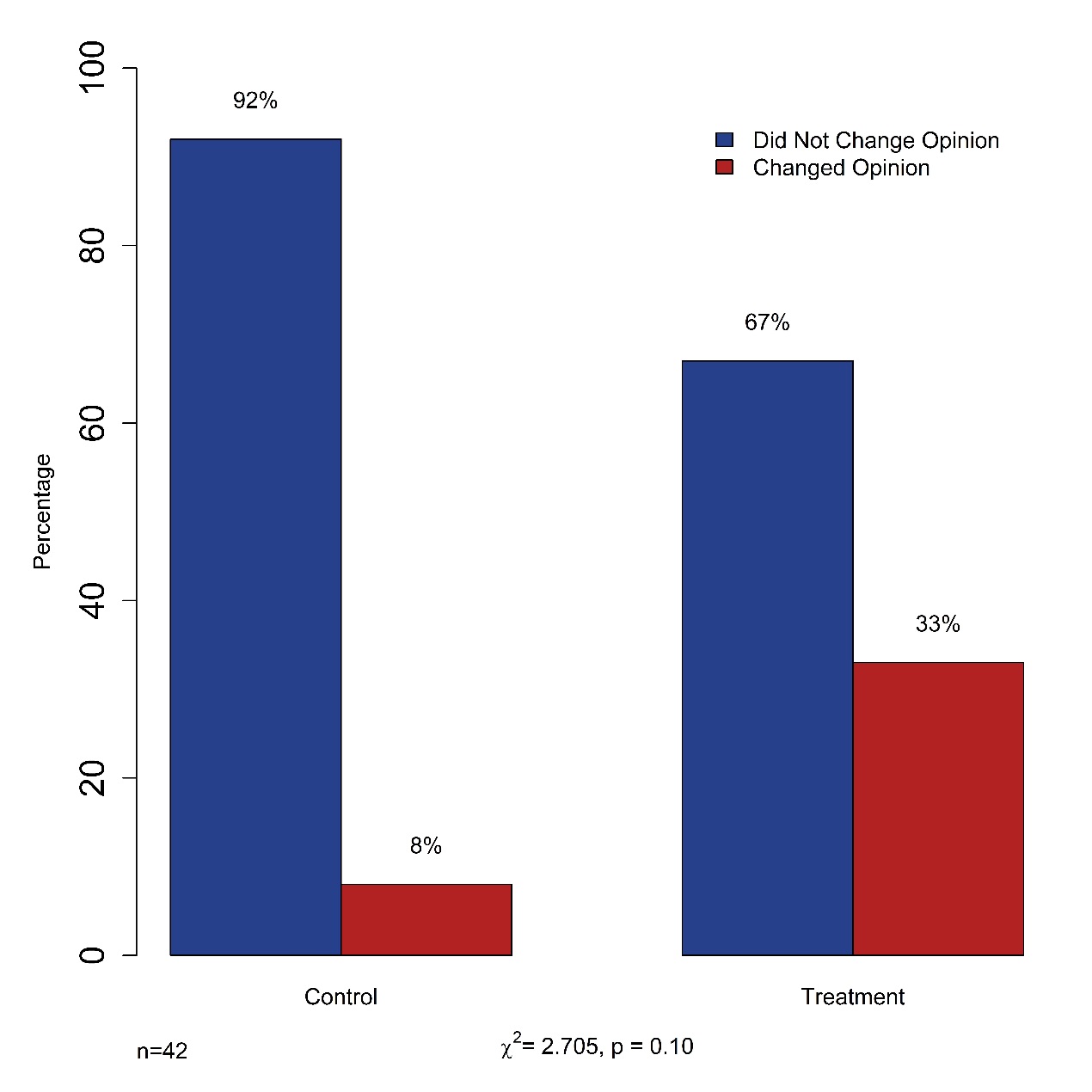

Supplement: S3 File — (DOCX) [file pone.0196600.s003.docx]
