# Supplementary material for: The effects of information and social conformity on opinion change
Source: PLoS One. 2018 May 2;13(5):e0196600. doi: 10.1371/journal.pone.0196600 (PMC5931497; doi:10.1371/journal.pone.0196600)
Supplement: S4 File — (DOCX) [file pone.0196600.s004.docx]

**S4 Appendix. Use of Deception in the Study Design**

Our interest in social conformity necessitated the use of deception in the study design. This approach is useful for political science experiments ([McDermott, 2013](#_ENREF_3)) and is necessary when informing the participants of the true purpose of the study would alter their behavior. In fact, we believe that participants would behave differently if they know that they would be discussing a political issue with a group of peers that uniformly did not share their opinion. We wanted as honest a response as possible from our participants; therefore we used the same deception format as the extant literature (e.g., [Asch, 1951](#_ENREF_1); [Hardy, 1957](#_ENREF_2)).

Of course, there is always concern that participants will discern the deception involved, particularly if they are familiar with psychological research or have participated in similar studies in the past. Fortunately, it appears that the deception used in this study was not identified. Participants were informed of the deception used in the research design during their debriefing in order to gather anecdotal evidence of its success. We received no indication from our participants during the debriefings that they were able to clearly discern the true purpose of the study and thus potentially manipulate its results. Indeed, only four participants indicated in their debriefing that they suspected some type of deception, but none were able to pinpoint the purpose of the study and the specific deception being used.

Given the feedback from the debriefings, we are confident that the deception worked according to the design. One participant’s comments encapsulate the types of comments we typically received after informing the participants about the deception used in the discussion session:

“Oh my god. So they were all in on it? I feel so joe schmoed right now. They were really good at that. I feel like those were their actual positions because they were really passionate about it.”

Many participants expressed appreciation for participating in the study even after learning about the deception. They generally found it to be a healthy and calm discussion. Furthermore, even after learning of the deception, participants told us that they learned a lot and had more thinking to do on the topic in light of that new information.

There were initially four confederates in each discussion group. Unfortunately, due to the length of the study, two confederates could not complete the entire study. For this reason, one additional confederate was recruited and trained and given the same talking points as the previous confederates, so that the groups would generally not fall below three confederates. However, there were two instances where one confederate did not show up for the discussion group. Therefore, there were sixteen discussion groups with four confederates, sixteen with three confederates, and two with two confederates. We found no significant differences based on the number of confederates.
