# Supplementary material for: The effects of information and social conformity on opinion change
Source: PLoS One. 2018 May 2;13(5):e0196600. doi: 10.1371/journal.pone.0196600 (PMC5931497; doi:10.1371/journal.pone.0196600)
Supplement: S5 File — (DOCX) [file pone.0196600.s005.docx]

**S5 Appendix: Breakdown of Opinion Change in the Treatment and Control Groups**

This supplemental information supports Fig 2 in the main body of the paper. It describes how we coded opinion change for both the treatment and control groups. As noted in the paper, we collected a participant’s opinion at multiple points during the experiment. For both groups, we have the initial opinion provided in the pre-test survey which asked participants “given what is known regarding the Jerry Sandusky incident at Penn State, the Board of Trustees was justified in firing Head Coach Joe Paterno?” Respondents answered using a five-point Likert scale ranging from Strongly Agree to Strongly Disagree.

For the control group, we then asked them to read the information sheet (see Supplement S1) from the treatment condition, as well as the arguments that are counter to their initial opinion (see Supplements S2a and S2b). Control group participants that answered “Neutral” to the initial opinion question were provided both sets of arguments. After reading through the material, we asked the control group participants again whether they believed Paterno should have been fired. This time, they answered the question in two parts. The first was either a “yes” or “no” and the second asked them the strength of their opinion from “neutral” to “strong.” We assigned treatment group participants to five sub-categories based on the possible opinions and opinion strengths for each set of questions. Fig A shows the percentage of the treatment group that fall into each sub-category. First, 54 percent of the treatment group provided a clear opinion (i.e., not neutral) to the initial opinion question and then provided the same clear opinion after reading the additional information. A total of 8 percent of the group responded to the information in some way. Four percent changed their opinion from one side to the other (Information Change), while the other four percent initially provided a neutral opinion, but then took a clear stance after reading the information (Information Decision). For the remaining 37 percent of the control group, some participants held the same opinion before and after the information, but it either gained or lost intensity. Specifically, 29 percent weakened their opinion and 8 percent strengthened it. We collapsed the two information categories into a single category of Opinion Change (8 percent) and collapsed the remaining three into No Opinion Change (92 percent).

**Fig A. Opinion changes for control group participants by subcategory**

**
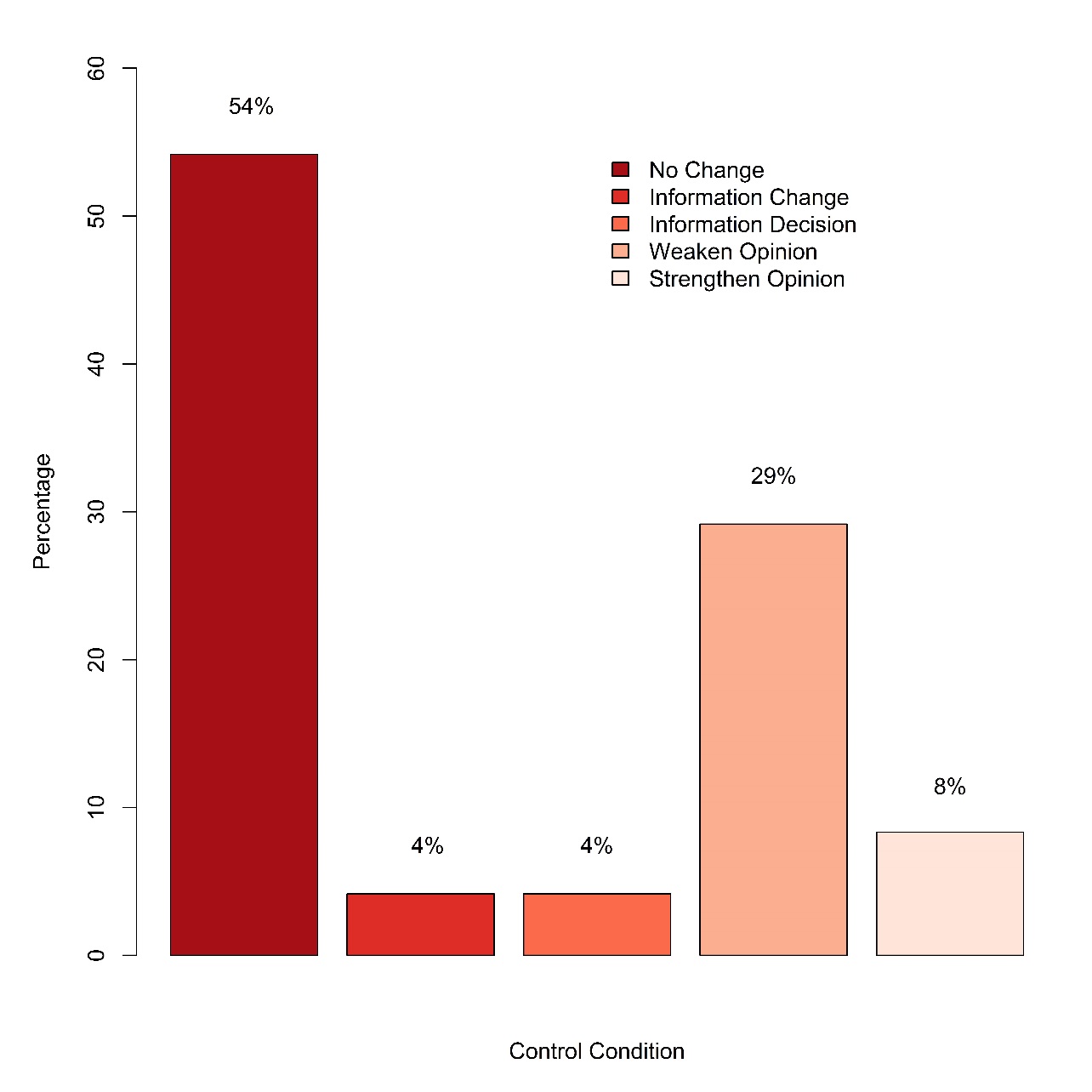
**

For the treatment group, we have three sets of recorded opinions. First, there is the initial opinion from the pre-test survey. Second, we have the yes or no answer provided after reading the information sheet and before starting the discussion period. Third, we have the “anonymous” ballot (yes or no) provided at the end of the discussion. We also have the verbal appearance of change coded from the video recordings, however since we are interested in actual opinion change for the main finding, we do not evaluate this for calculating overall change for Fig 3 in the paper. Given these three different opinions, we again have five sub-categories, but they are slightly different given the lack of intensity measure for the final vote. Fig B displays the percentages of the treatment group falling into each sub-category. The largest group, once again, is the one that did not change their opinion at any time (44 percent). However, like the control group, there were some individuals that changed or decided their opinion based solely on the information presented before the discussion. Three percent changed from one clear opinion to another between the pre-test survey and the pre-discussion voice vote (Information Change) and fifteen percent moved from a neutral position to a choice of yes or no that they then maintained in the secret ballot (Information Decision). Relatedly, fifteen percent of the treatment group started as neutral in the pre-test, took a position on the initial ballot, and then changed that position after the discussion (Information Decision and Discussion Change). Finally, 24 percent of the treatment group had the same opinion in the pre-test survey and the initial voice vote, but changed that opinion after the discussion. Given that we are interested in the extent to which individual changed their opinion during the treatment group discussion, we merged the first three categories into No Opinion Change (62 percent) and Opinion Change (38 percent).^[[1]](#footnote-1)^

**Fig B. Opinion changes for treatment group participants by sub-category**


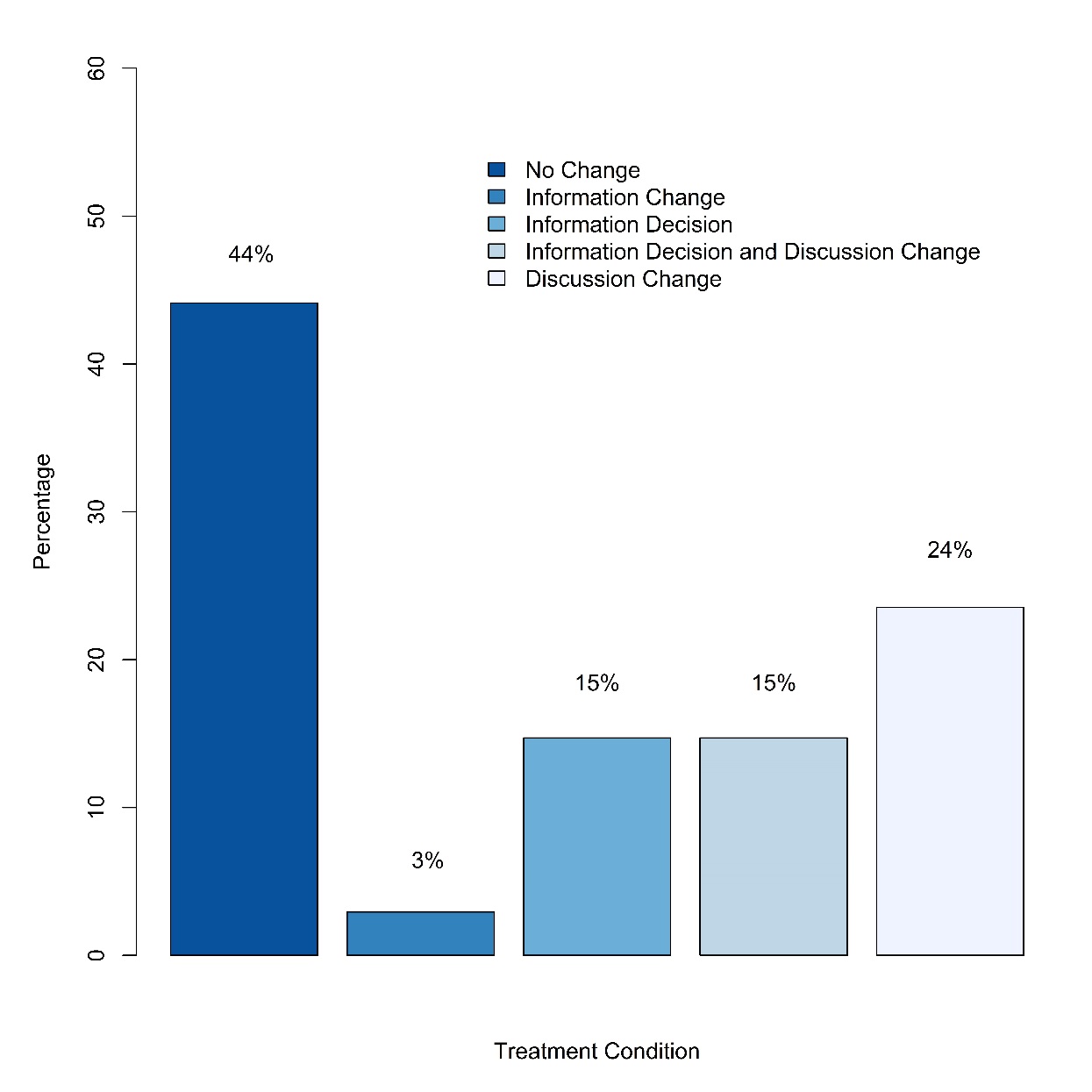


1. Slight differences from totals based on Fig B are the result of rounding. [↑](#footnote-ref-1)
